# Distressing dreams, cognitive decline, and risk of dementia: A prospective study of three population-based cohorts

**DOI:** 10.1016/j.eclinm.2022.101640

**Published:** 2022-09-21

**Authors:** Abidemi I. Otaiku

**Affiliations:** a Department of Neurology, Birmingham City Hospital, Birmingham, UK; b Centre for Human Brain Health, University of Birmingham, Birmingham, UK

**Keywords:** Dreaming, Cognitive decline, Dementia, Nightmares

## Abstract

**Background:**

Distressing dreams are associated with faster cognitive decline and increased dementia risk in people with Parkinson's disease (PD). Whether distressing dreams might be associated with cognitive decline and dementia in people without PD is unknown. This study investigated the association between self-reported distressing dream frequency and the risk of cognitive decline and incident dementia in community-dwelling men and women without cognitive impairment or PD.

**Methods:**

Risk of cognitive decline was evaluated in 605 middle-aged adults (mean age = 50 years [IQR 44–57]; 55·7% female) from the Midlife in the United States (MIDUS) study, who were cognitively normal at baseline, and were followed-up for a maximum of 13 years (IQR 9–10). Cognitive decline was defined as having an annual rate of decline in global cognitive function (measured using five cognitive tests) ≥ 1 standard deviation faster than the mean decline rate from baseline to follow-up. Risk of incident all-cause dementia was evaluated in 2600 older adults (mean age = 83 years [IQR 81–84]; 56·7% female) pooled from the Osteoporotic Fractures in Men Study (MrOS) and the Study of Osteoporotic Fractures (SOF), who were dementia-free at baseline, and were followed-up for up a maximum of 7 years (IQR 4–5). Incident dementia was based on doctor-diagnosis. Frequency of distressing dreams was assessed in all cohorts at baseline (January 2002 – March 2012) using item 5h of the Pittsburgh Sleep Quality Index. The association between self-reported distressing dream frequency (“never”, “less than weekly”, “weekly”) and later cognitive outcomes, was evaluated using multivariable logistic regression in both the middle-aged and pooled older adult cohorts.

**Findings:**

After adjustment for all covariates, a higher frequency of distressing dreams was linearly and statistically significantly associated with higher risk of cognitive decline amongst middle-aged adults (*P* for trend = 0·016), and higher risk of incident all-cause dementia amongst older adults (*P* for trend <0·001). Compared with middle-aged adults who reported having no distressing dreams at baseline, those who reported having weekly distressing dreams had a 4-fold risk of experiencing cognitive decline (adjusted odds ratio [aOR] = 3·99; 95% CI: 1·07, 14·85). Amongst older adults, the difference in dementia risk was 2·2-fold (aOR = 2·21; 95% CI: 1·35, 3·62). In sex-stratified analyses, the associations between distressing dreams and both cognitive outcomes were only statistically significant amongst men.

**Interpretation:**

Distressing dreams predict cognitive decline and all-cause dementia in middle-aged and older adults without cognitive impairment or PD - especially amongst men. These findings may help to identify individuals at risk of dementia and could facilitate early prevention strategies.

**Funding:**

The study received no external funding.

**Research in context**

**Evidence before this study**

A PubMed search was conducted using the search string: ((‘nightmares’ OR ‘bad dreams’ OR ‘dream content’)) AND ((‘dementia’ OR ‘Alzheimer's disease’ OR ‘cognitive decline’)); to identify primary research studies published in any language up until July 24, 2022. Previous longitudinal studies have shown that distressing dreams (nightmares and bad dreams) predict future cognitive decline and dementia in people with Parkinson's disease (PD). However, no study has evaluated whether distressing dreams might also predict cognitive decline and dementia in adults without PD.

**Added value of this study**

This prospective, longitudinal analysis of three population-based cohorts from the USA, has shown for the first time that a higher frequency of distressing dreams in community-dwelling adults without cognitive impairment or PD, is positively associated with faster rates of cognitive decline during midlife, and increased risk of developing dementia during later life. Moreover, these associations were found to be strongest in men.

**Implications of all the available evidence**

Adults who report having distressing dreams during middle and older age, may experience accelerated cognitive decline and are at increased risk of developing dementia. Screening for distressing dreams may help to identify individuals at risk of dementia and could facilitate early prevention strategies.

Alt-text: Unlabelled box

## Introduction

Nightmares are common in the general population. Approximately 5% of adults experience nightmares weekly,^1^^,^^2^ with a further 12–40% experiencing them monthly.^1^^,^^2^ These percentages are likely even higher when considering bad dreams alongside nightmares.^3^ Given the ubiquity of bad dreams and nightmares (distressing dreams) in the adult population, it is surprising that their clinical significance remains largely unknown.

However, in recent years, the relationship between distressing dreams and clinical outcomes in people with Parkinson's disease (PD), has received increasing attention.^4^ Three studies have shown that a higher frequency of distressing dreams in people with nondemented PD, is prospectively associated with faster rates of cognitive decline, and increased risk of developing dementia over time.^4^, ^5^, ^6^ Thus, these findings raise the intriguing possibility that distressing dreams might also be associated with faster cognitive decline and increased dementia risk in adults without PD.

While recent studies in community-dwelling adults have shown that distressing dreams become more frequent with advancing age,^2^^,^^7^ and are associated with poor cognitive function cross-sectionally^8^^,^^9^; no study has investigated whether they may be associated with cognitive decline and dementia longitudinally.

This study investigated the hypothesis that a higher frequency of distressing dreams in middle-aged and older adults without cognitive impairment or PD (measured using a self-report questionnaire), would be positively associated with faster rates of cognitive decline, and increased risk of developing dementia over time. This theory was tested using longitudinal data from three population-based cohorts from the USA.^10^, ^11^, ^12^

## Methods

### Study populations

#### Midlife in the United States (MIDUS) study

The middle-aged cohort in this analysis comprised community-dwelling men and women from the Midlife in the United States (MIDUS) study.^10^ MIDUS is an ongoing longitudinal study, beginning in 1995, which has included over 7000 young, middle-aged and older adults from the USA, who were recruited through a nationally representative random-digit dialling sampling strategy, in addition to specific subsamples consisting of siblings and twins.

For the present analysis, baseline was defined as MIDUS Wave 2 (January 2004 – September 2009), during which 1016 adults participated in both the Biomarker Subproject (during which the Pittsburgh Sleep Quality Index [PSQI]^13^ was administered) and the Cognitive Subproject (during which detailed cognitive assessments were first administered). From the 1016 adults participating in both subprojects, only those who were middle-aged (35–64 years) at baseline, completed the questionnaire item on distressing dreams (99·9%), and had baseline cognitive function data available, were included in this analysis (*n* = 745). Of these participants, those with probable cognitive impairment (defined as global cognitive function *z* scores below 1 standard deviation [SD] of the MIDUS 2 population mean)^14^ or self-reported PD at baseline, were excluded (*n* = 53). Participants who died or ended participation before the MIDUS 3 Cognitive Subproject (July 2013 – March 2017; *n =* 70) or had missing data for the repeat cognitive assessments (*n =* 17, 2·5%), were also excluded. This left a total of 605 men and women in the middle-aged analytic cohort.

#### The study of osteoporotic fractures (SOF)

The older women in the present analysis were from SOF.^11^ SOF is an observational, longitudinal cohort study that enrolled 10366 community-dwelling women aged 65 years or over at four clinical centres in the USA, including: Baltimore, Minneapolis, Portland and Pittsburgh. In order to participate, the women needed to be able to walk without assistance and must not have had a bilateral hip replacement.

For this analysis, baseline for SOF participants was defined as Visit 8 (January 2002 - April 2004), during which the PSQI was administered. From the 4261 women participating in Visit 8, only those who completed the questionnaire item on distressing dreams (95·7%), and had data available to determine baseline cognitive status, were included in this analysis (*n =* 3151). Of these participants, those with probable cognitive impairment (self-reported doctor diagnosis of dementia and/or Mini Mental State Examination [MMSE]^15^ score <25) or self-reported PD at baseline, were excluded (*n =* 367). Women who died or ended participation before follow-up (Visit 9 [January 2007 – June 2008]; *n =* 1231) or had missing data for incident dementia (*n =* 78, 5·0%), were also excluded. This left a total of 1475 women for inclusion in the pooled older adult cohort.

#### The osteoporotic fractures in men study (MrOS)

The older men in the present analysis were from MrOS.^12^ MrOS is an observational, longitudinal cohort study that enrolled 5994 community-dwelling men aged 65 years or over at six clinical centres in the USA, including: Birmingham, Minneapolis, Palo Alto, Pittsburgh, Portland, and San Diego. In order to participate, the men needed to be able to walk without assistance and must not have had a bilateral hip replacement.

For this analysis, baseline for MrOS participants was defined as either Sleep Visit 1 (December 2003 – March 2005) or Sleep Visit 2 (November 2009 – March 2012), during which the PSQI was administered. From the 3135 men participating in Sleep Visit 1, only those who were a similar age to the women in SOF at baseline (79+), completed the questionnaire item on distressing dreams (99·9%), and had data available to determine baseline cognitive status, were included in this analysis (*n =* 1050). Of these participants, those with probable cognitive impairment (dementia medication use and/or Modified Mini Mental State Examination [3MS]^16^ score <80) or self-reported PD at baseline, were excluded (*n =* 100). Men who died or ended participation before the first follow-up after baseline (Visit 3 [March 2007 – March 2009]; *n =* 145) or had missing data for incident dementia (*n =* 11, 1·4%), were also excluded. This left 794 men from Sleep Visit 1 for inclusion in the pooled older adult cohort. Men who were excluded from Sleep Visit 1 for being below the age of 79, but met all other eligibility criteria, were subsequently included at Sleep Visit 2 (*n =* 331). Therefore, a total of 1125 men from the two baseline visits were included in the pooled older adult cohort. To enable comparisons between MrOS and SOF, only the first 7 years of follow-up in MrOS (up to 2016) were considered in this analysis.

### Distressing dreams

Participants in all cohorts completed the PSQI at baseline, a validated self-report questionnaire for assessing habitual sleep quality and disturbances.^13^ Distressing dream frequency was assessed using item 5h of the PSQI: “During the past month, how often have you had trouble sleeping because you have bad dreams?”. No definition of bad dreams was provided. The options included: (0) “not during the past month, (1) “Less than once a week”, (2) “once or twice a week” and (3) “three or more times a week”. In this analysis, the latter two categories were combined to create a single category “once a week or more”, to be consistent with previous studies.^1^^,^^9^

### Cognitive decline

In MIDUS, cognitive function was assessed both at baseline and at follow-up using the Brief Test of Adult Cognition by Telephone (BTACT).^17^^,^^18^ The BTACT includes subtests that measure episodic memory (immediate and delayed free recall of 15 words), working memory (backward digit span), verbal fluency (category fluency), inductive reasoning (number series completion), and processing speed (backward counting task).^17^ The individual test scores were *z*-scored (mean = 0; SD = 1) according to means and standard deviations of the MIDUS 2 full sample. The five *z* scores were then summed and restandardised to yield a global (composite) score. A higher composite *z* score represents better global cognitive functioning. This approach has been used to calculate *z* scores of global cognitive function in the MIDUS study^18^ and elsewhere.^19^

An annualised rate of change for global cognitive function was calculated for each participant by subtracting their composite *z* score at baseline, from their composite *z* score at follow-up, and then dividing by the length of follow-up in years:

*Annualised rate of change = (composite z score at follow up − composite z score at baseline)/(length of follow-up in years).*

Cognitive decline at follow-up was defined as having an annual rate of decline in global cognitive function ≥ 1 SD faster than the mean rate of change. This cut-off corresponds to a clinically significant decline that is faster than expected for normal cognitive ageing.^20^

### Incident dementia

At follow-up in MrOS and SOF, participants were asked to report whether they had ever been diagnosed with dementia by a doctor. In addition, participants were asked to bring in all prescription medications they had used in the past 30 days, including dementia medications. Dementia medication use was verified by clinic staff.

Incident all-cause dementia was defined in this study as either use of medication for treating dementia or self-reported doctor diagnosis of dementia.

### Covariates

Potential confounders were chosen based on *a priori* knowledge of factors associated with nightmares, cognitive decline, and dementia risk, as well as previous studies.^1^^,^^7^^,^^21^, ^22^, ^23^ These included: age in years (35-39, 40-49, 50-59, 60-64, 79-89, 90+), sex (male, female), race (white, non-white), educational qualifications (college degree or equivalent, high school or equivalent, none), cohabitation status (lives alone, cohabiting), current smoking (yes/no), alcohol intake (drinks/month, <1, ≥1), self-rated health (good/excellent, poor/fair), physical inactivity (yes/no), body mass index (BMI; continuous), habitual sleep duration (continuous), frequency of sleep onset insomnia (times/week, 0, <1, 1-2, ≥3), frequency of sleep maintenance insomnia (times/week, 0, <1, 1-2, ≥3), daytime sleepiness (continuous), history of doctor-diagnosed diabetes mellitus (yes/no), history of doctor-diagnosed hypertension (yes/no), history of doctor-diagnosed stroke (yes/no), current depression (MIDUS: depressive symptoms, continuous; MrOS and SOF: clinically significant depression, yes/no), current anxiety (MIDUS: trait anxiety, continuous; MrOS and SOF: clinically significant anxiety, yes/no), medication use (yes/no) and baseline cognitive function (MIDUS: continuous; MrOS and SOF: normal cognition/possible mild cognitive impairment [MCI]). In addition, MIDUS included current psychosocial stress (continuous) and MrOS and SOF included history of doctor-diagnosed sleep apnoea (yes/no/don't know), and history of doctor-diagnosed non-apnoea sleep disorders (yes/no/don't know).

Age, sex, race, educational qualifications, cohabitation status, alcohol intake, smoking status, habitual sleep duration, history of doctor-diagnosed medical conditions, physical activity, and self-rated health, were self-reported at baseline. In MIDUS, physical inactivity was defined as less than three days a week of any physical activity lasting 20 minutes or more. In MrOS and SOF, physical inactivity was defined as leaving the house less than once per week. BMI was calculated as weight divided by height (kg/m^2^). Sleep onset insomnia and sleep maintenance insomnia were assessed using items 5a and 5b of the PSQI respectively (scores ranging from 0 “not during the past month”, to 3 “three or more times a week”).^13^ Daytime sleepiness was assessed using item 7 of the PSQI (scores ranging from 0 “no problem at all”, to 3 “very big problem”).^13^ Depressive symptoms were evaluated using the Center for Epidemiologic Studies Depression Scale in MIDUS,^24^ and the Geriatric Depression Scale in MrOS and SOF (with scores ≥6 indicating clinically significant depression).^25^ Anxiety symptoms were evaluated using the Spielberger Trait Anxiety Inventory in MIDUS,^26^ and the Goldberg Anxiety Scale in MrOS and SOF (with scores ≥5 indicating clinically significant anxiety).^27^ Global cognitive function was measured using the composite cognitive *z* score in MIDUS (continuous), the MMSE in SOF (with scores <28 indicating possible MCI),^15^ and the 3MS in MrOS (with scores <89 indicating possible MCI).^16^ Psychosocial stress was measured in MIDUS using the Perceived Stress Scale.^28^ Medication use was defined as: antidepressants, benzodiazepines or hypnotics.^29^ The percentage of participants with missing information for covariates was small (MIDUS: 3%; MrOS and SOF combined: 2%). Indicator variables were used for missing information for categorical covariates, and median imputation was used for missing continuous covariates.

### Statistical analysis

Characteristics of the participants at baseline in the middle-aged and pooled older adult cohorts, stratified by distressing dream frequency, were compared using χ^2^ tests for categorical variables, ANOVA for normally distributed continuous variables, and Kruskal–Wallis tests for nonnormally distributed variables.

Multivariable logistic regression was used to obtain odds ratios (ORs) and 95% confidence intervals (CIs) to determine the association between distressing dream frequency and risk of: (i) cognitive decline (middle-aged cohort), and (ii) incident all-cause dementia (pooled older adult cohort). In all analyses, distressing dream frequency was modelled as both a categorical variable (referent group “not during the past month”) and as a continuous variable (to obtain a *P* value for linear trend).

All analyses were adjusted for possible confounders. Model 1 was minimally adjusted for age, sex and baseline cognitive function. Model 2 further adjusted for race, education, cohabitation status, depression, anxiety, sleep onset insomnia, sleep maintenance insomnia, daytime sleepiness, habitual sleep duration, diabetes, hypertension, stroke, physical inactivity, BMI, self-rated health, smoking status, alcohol intake, and medication use. The primary analyses were conducted for both sexes combined, and the secondary analyses were stratified by sex.

In sensitivity analyses, the regression models were repeated after: (i) further adjusting for psychosocial stress (middle-aged cohort only), (ii) further adjusting for doctor-diagnosed sleep disorders (older adult cohort only), (iii) excluding participants with possible MCI at baseline (older adult cohort only), and (iv) excluding participants who developed PD by the end of the follow-up period (both cohorts).

Statistical testing was performed two-sided at *P* <0·05. All analyses were performed using SPSS version 28 (IBM Corp., Armonk, NY).

### Ethical considerations

All participants provided written informed consent. The original studies were approved by the institutional review boards involved with MIDUS, SOF and MrOS. The present study received approval from the University of Birmingham (Ref No ERN_21-1463).

### Role of the funding source

There was no funding source for this study. AIO had full access to the dataset and had final responsibility for the decision to submit for publication.

## Results

Baseline characteristics of the middle-aged cohort (*n =* 605; mean [SD] age = 50·3 [7·9] years; 55·7% female; 6·6% non-white) and older adult cohort (*n =* 2600; mean [SD] age = 82·9 [2·9] years; 56·7% female; 2·8% non-white) stratified by distressing dream frequency, are presented in Tables 1 and 2 respectively. In both cohorts, approximately 77% of the participants reported no distressing dreams in the previous month (middle-aged: 72·9%; older adult: 80·0%); approximately 17% reported having distressing dreams less than once a week (middle-aged: 21·2%; older adult: 13·1%); and approximately 6% reported having distressing dreams at least once a week (middle-aged: 6·0%; older adult: 6·9%).

**Table 1:** Baseline characteristics of the middle-aged cohort by distressing dream frequency.

	Distressing dream frequency (past month)			
Characteristic	Never	<1/week	≥1/week	*P* value
*N*	441	128	36	
Age, yrs, *n* (%)				0·66
35 – 39	44 (10·0)	14 (10·9)	6 (16·7)	
40 – 49	161 (36·5)	46 (35·9)	12 (33·2)	
50 – 59	168 (38·1)	55 (43·0)	13 (36·1)	
60 – 64	68 (15·4)	13 (10·2)	5 (13·9)	
Sex, *n* (%)				0·03
Male	209 (47·4)	48 (37·5)	11 (30·6)	
Female	232 (52·6)	80 (62·5)	25 (69·4)	
Race, *n* (%)				0·40
White	415 (94·1)	118 (92·2)	32 (88·9)	
Non-white	26 (5·9)	10 (7·8)	4 (11·1)	
Education, *n* (%)				0·25
Less than high school	4 (0·9)	3 (2·3)	1 (2·8)	
High School	162 (36·7)	57 (44·5)	15 (41·7)	
College	275 (62·4)	68 (53·1)	20 (55·6)	
Cohabitation status, *n* (%)				0·40
Lives alone	86 (19·5)	28 (21·9)	11 (30·6)	
Cohabiting	351 (79·6)	99 (77·3)	24 (66·7)	
Missing	4 (0·9)	1 (0·8)	1 (2·8)	
Cognitive function, z-score	0·50 ± 0·8	0·42 ± 0·8	0·33 ± 0·7	0·42
Sleep onset insomnia, times/wk, *n* (%)				<0·001
0	231 (52·4)	42 (32·8)	12 (33·3)	
<1	140 (31·7)	44 (34·4)	8 (22·2)	
1-2	45 (10·2)	21 (16·4)	8 (22·2)	
≥3	24 (5·4)	21 (16·4)	8 (22·2)	
Missing	1 (0·2)	0 (0·0)	0 (0·0)	
Sleep maintenance insomnia, times/wk, *n* (%)				<0·001
0	98 (22·2)	13 (10·2)	3 (8·3)	
<1	123 (27·9)	28 (21·9)	5 (13·9)	
1-2	103 (23·4)	34 (26·6)	11 (30·6)	
≥3	117 (26·5)	53 (41·4)	16 (44·4)	
Missing	0 (0·0)	0 (0·0)	1 (2·8)	
Habitual sleep duration (hrs)^a^	6·9 ± 1·1	6·7 ± 1·0	7·1 ± 1·3	0·21
Daytime sleepiness^b^	0·28 ± 0·6	0·30 ± 0·6	0·44 ± 0·8	0·43
Depressive symptoms^c^	6·7 ± 6·9	8·7 ± 7·3	15·3 ± 12·5	<0·001
Trait anxiety^d^	31·9 ± 8·1	35·5 ± 8·9	39·9 ± 10·5	<0·001
Psychosocial stress^e^	20·9 ± 6·2	23·0 ± 5·5	26·2 ± 7·4	<0·001
Hypertension, *n* (%)				0·18
Yes	112 (25·4)	38 (29·7)	15 (41·7)	
No	325 (73·7)	90 (70·3)	21 (58·3)	
Missing	4 (0·9)	0 (0·0)	0 (0·0)	
Diabetes, *n* (%)				0·98
Yes	34 (7·7)	10 (7·8)	3 (8·3)	
No	406 (92·1)	118 (92·2)	33 (91·7)	
Missing	1 (0·2)	0 (0·0)	0 (0·0)	
Stroke, *n* (%)				0·84
Yes	4 (0·9)	1 (0·8)	0 (0·0)	
No	437 (99·1)	127 (99·2)	36 (100·0)	
Self-rated health, *n* (%)				<0·001
Good/excellent	421 (95·5)	112 (87·5)	30 (83·3)	
Poor/fair	20 (4·5)	16 (12·5)	6 (16·7)	
Physical inactivity				0·41
Yes	88 (20·0)	23 (18·0)	4 (11·1)	
No	353 (80·0)	105 (82·0)	32 (88·9)	
BMI (kg/m^2^)^f^	29·0 ± 5·9	29·7 ± 6·7	30·1 ± 5·3	0·36
Medication use, *n* (%)				<0·001
Yes	83 (19·3)	39 (30·5)	22 (61·3)	
No	355 (80·5)	89 (69·5)	14 (38·9)	
Missing	1 (0·2)	0 (0·0)	0 (0·0)	
Alcohol intake, drinks/month, *n* (%)				0·90
<1	144 (32·7)	41 (32·0)	13 (36·1)	
≥1	297 (67·3)	87 (68·0)	23 (63·9)	
Current smoking, *n* (%)				0·11
Yes	389 (11·8)	105 (18·0)	29 (19·4)	
No	52 (88·2)	23 (82·0)	7 (80·6)	

Abbreviations: BMI, body mass index; SD, standard deviation.

Plus-minus values are means ± SD

a Data was missing for 2 participants.

b Pittsburgh Sleep Quality Index item 7. Scores range from 0 (no problem at all) to 4 (a very big problem).

c Center for Epidemiologic Studies Depression Scale. Scores range from 0 - 60, with higher scores indicating more severe depressive symptoms. Data was missing for 2 participants.

d Spielberger Trait Anxiety Inventory. Scores range from 0 - 80, with higher scores indicating higher trait anxiety. Data was missing for 1 participant.

e Perceived Stress Scale. Scores range from 0 - 40, with higher scores indicating higher levels of psychosocial stress. Data was missing for 2 participants.

f Data was missing for 1 participant.

**Table 2:** Baseline characteristics of the older adult cohort by distressing dream frequency.

	Distressing dream frequency (past month)			
Characteristic	Never	<1/week	≥1/week	*P* value
*N*	2079	341	180	
Age, yrs, *n* (%)				0·58
79-89	2009 (96·6)	332 (97·4)	171 (95·0)	
90+	67 (3·2)	9 (2·6)	9 (5·0)	
Missing	3 (0·1)	0 (0·0)	0 (0·0)	
Sex, *n* (%)				<0·001
Male	844 (40·6)	185 (54·3)	96 (53·3)	
Female	1235 (59·4)	156 (45·7)	84 (46·7)	
Race, *n* (%)				0·052
White	2029 (97·6)	325 (95·3)	174 (96·7)	
Non-white	50 (2·4)	16 (4·7)	6 (3·3)	
Education, *n* (%)				0·72
Less than high school	219 (10·5)	32 (9·4)	22 (12·2)	
High School	1123 (54·0)	174 (51·0)	95 (52·8)	
College	734 (35·3)	135 (39·6)	63 (35·0)	
Missing	3 (0·1)	0 (0·0)	0 (0·0)	
Cohabitation status, *n* (%)				0·16
Lives alone	997 (48·0)	150 (44·0)	76 (42·2)	
Cohabiting	1082 (52·0)	191 (56·0)	104 (57·8)	
Cognitive function, *n* (%)				0·44
Possible MCI	404 (19·4)	69 (20·2)	42 (23·3)	
Normal cognition	1675 (80·6)	272 (79·8)	138 (76·7)	
Sleep onset insomnia, times/wk, *n* (%)				<0·001
0	1024 (49·3)	118 (34·6)	58 (32·2)	
<1	477 (22·9)	106 (31·1)	39 (21·7)	
1-2	283 (13·6)	56 (16·4)	38 (21·1)	
≥3	294 (14·1)	61 (17·9)	45 (25·0)	
Missing	1 (0·0)	0 (0·0)	0 (0·0)	
Sleep maintenance insomnia, times/wk, *n* (%)				<0·001
0	419 (20·2)	33 (9·7)	7 (3·9)	
<1	238 (11·4)	38 (11·1)	12 (6·7)	
1-2	342 (16·5)	51 (15·0)	18 (10·0)	
≥3	1080 (51·9)	219 (64·2)	142 (78·9)	
Missing	0 (0·0)	0 (0·0)	1 (0·6)	
Habitual sleep duration (hrs)	7·1 ± 1·2	7·0 ± 1·2	7·0 ± 1·4	0·82
Daytime sleepiness^a^	0·13 ± 0·5	0·18 ± 0·5	0·24 ± 0·6	0·003
Sleep apnoea, *n* (%)				<0·001
Yes	63 (3·0)	19 (5·6)	13 (7·2)	
No	1985 (95·5)	313 (91·8)	160 (88·9)	
Unknown	31 (1·5)	9 (2·6)	7 (3·9)	
Non-apnoea sleep disorder, *n* (%)				0·01
Yes	106 (5·1)	31 (9·1)	14 (7·8)	
No	1954 (94·0)	305 (89·4)	162 (90·0)	
Unknown	19 (0·9)	25 (1·5)	4 (2·2)	
Depression, *n* (%)				0·001
Yes	140 (6·7)	31 (9·1)	27 (15·0)	
No	1937 (93·2)	310 (90·9)	153 (85·0)	
Missing	2 (0·1)	0 (0·0)	0 (0·0)	
Anxiety, *n* (%)				<0·001
Yes	178 (8·6)	45 (13·2)	46 (25·6)	
No	1898 (91·3)	295 (86·5)	134 (74·4)	
Missing	3 (0·1)	1 (0·3)	0 (0·0)	
Hypertension, *n* (%)				0·079
Yes	1120 (53·9)	198 (58·1)	110 (61·1)	
No	959 (46·1)	143 (41·9)	70 (38·9)	
Diabetes, *n* (%)				0·81
Yes	212 (10·2)	38 (11·1)	17 (9·4)	
No	1867 (89·8)	303 (88·9)	163 (90·6)	
Stroke, n (%)				0·42
Yes	152 (7·3)	25 (7·3)	18 (10·0)	
No	1927 (92·7)	316 (92·7)	162 (90·0)	
Self-rated health, *n* (%)				0·003
Good/excellent	1757 (84·5)	278 (81·5)	135 (75·0)	
Poor/fair	322 (15·5)	63 (18·5)	45 (25·0)	
Physical inactivity				0·12
Yes	109 (5·2)	11 (3·2)	13 (7·2)	
No	1970 (94·8)	330 (96·8)	167 (92·8)	
BMI (kg/m^2^)^b^	26·7 ± 4·0	27·0 ± 3·8	27·0 ± 3·5	0·15
Medication use, *n* (%)				<0·001
Yes	295 (14·2)	57 (14·1)	52 (28·9)	
No	1784 (85·8)	284 (83·0)	128 (71·1)	
Missing	0 (0·0)	1 (0·3)	0 (0·0)	
Alcohol intake, drinks/month, *n* (%)				0·65
<1	974 (46·8)	156 (45·7)	78 (43·3)	
≥1	1102 (53·0)	184 (54·0)	101 (56·1)	
Missing	3 (0·1)	1 (0·3)	1 (0·6)	
Current smoking, *n* (%)				0·094
Yes	25 (1·2)	2 (0·6)	5 (2·8)	
No	2054 (98·8)	339 (99·4)	175 (97·2)	

Abbreviations: BMI, body mass index; MCI, mild cognitive impairment; SD, standard deviation.

Plus-minus values are means ± SD

a Pittsburgh Sleep Quality Index item 7. Scores range from 0 (no problem at all) to 4 (a very big problem).

b Data was missing for 36 participants.

Distressing dreams were more frequent amongst women in the middle-aged cohort (Table 1), but were more frequent amongst men in the older cohort (Table 2). In both cohorts, compared with participants who reported having no distressing dreams at baseline, those who had distressing dreams tended to be more depressed, more anxious, had more sleep problems, had worse self-rated health, and were more likely to use medications that can affect dreaming. There were no differences in regard to baseline cognitive function.

In the middle-aged cohort, the mean rate of change in global cognitive function during the 13-year follow-up period (IQR 9–10), was a decline of 0·024 standardised score units per year. 90 (14·9%) participants met the criteria for cognitive decline at follow-up. In the fully adjusted model (Table 3), a higher frequency of distressing dreams was linearly and statistically significantly associated with higher risk of cognitive decline (*P* for trend = 0·016). Compared with participants who reported having no distressing dreams at baseline, those who reported having weekly distressing dreams had a 4-fold risk of experiencing cognitive decline (adjusted OR [aOR] = 3·99; 95% CI: 1·07, 14·85; *P*=0·039).

**Table 3:** Risk of cognitive decline in the middle-aged cohort by baseline distressing dream frequency (OR and 95% CI).

	Distressing dream frequency (past month)			*P* for linear trend
Analysis	Never	<1/week	≥1/week	
Cognitive decline [*n* (%)]	62 (14·1)	22 (17·2)	6 (16·7)	
*N*	441	128	36	
Main Analysis				
Age-, Sex and Cognition Adjusted Model	1 [reference]	1·77 (0·91, 3·46)	2·61 (0·87, 7·78)	0·027*
Fully adjusted Model^a^	1 [reference]	1·97 (0·91, 4·24)	3·99 (1·07, 14·85)*	0·016*
Stratified Analysis^b^				
Men	1 [reference]	4·05 (0·99, 16·55)	4·45 (0·44, 45·06)	0·045*
Women	1 [reference]	1·30 (0·42, 4·07)	4·03 (0·56, 29·12)	0·231
Sensitivity Analysis 1^c^	1 [reference]	1·95 (0·90, 4·22)	4·12 (1·09, 15·53)*	0·015*
Sensitivity Analysis 2^d^	1 [reference]	1·97 (0·91, 4·25)	3·98 (1·07, 14·84)*	0·016*

Abbreviations: OR, odds ratios; CI, confidence interval.

a Adjusted for age, sex, baseline cognitive function, race, education, cohabitation status, sleep onset insomnia, sleep maintenance insomnia, daytime sleepiness, habitual sleep duration, depression, anxiety, diabetes, hypertension, stroke, self-rated health, physical inactivity, BMI, smoking status, alcohol intake, and medication use.

b Analysis stratified by sex, with adjustment for covariates in the fully adjusted model.

c Sensitivity analysis 1 further adjusting for psychosocial stress, in addition to covariates in the fully adjusted model.

d Sensitivity analysis 2 excluded participants who developed PD by the end of the follow-up period (*n*=1). This analysis was adjusted for covariates in the full model. *P < 0·05. ^⁎⁎^*P* < 0·01. ^⁎⁎⁎^*P* < 0·001.

In the older adult cohort, 235 (9·0%) participants were diagnosed with clinical dementia during the 7-year follow-up period (IQR 4–5). In the fully adjusted model (Table 4), a higher frequency of distressing dreams was linearly and statistically significantly associated with higher risk of developing dementia (*P* for trend <0·001). Compared with participants who reported having no distressing dreams at baseline, those who reported having weekly distressing dreams had a 2·2-fold risk of developing dementia (aOR = 2·21; 95% CI: 1·35, 3·62; *P*=0·002).

**Table 4:** Risk of incident all-cause dementia in the older adult cohort by baseline distressing dream frequency (OR and 95% CI).

	Distressing dream frequency (past month)			*P* for linear trend
Analysis	Never	<1/week	≥1/week	
Dementia cases [*n* (%)]	178 (8·6)	32 (9·4)	25 (13·9)	
*N*	2079	341	180	
Main Analysis				
Age-, Sex and Cognition Adjusted Model	1 [reference]	1·21 (0·81, 1·82)	1·85 (1·16, 2·94)**	0·010**
Fully adjusted Model^a^	1 [reference]	1·41 (0·93, 2·13)	2·21 (1·35, 3·62)**	<0·001***
Stratified Analysis^b^				
Men	1 [reference]	1·50 (0·70, 3·18)	4·67 (2·16, 10·11)***	<0·001***
Women	1 [reference]	1·41 (0·84, 2·35)	1·41 (0·70, 2·83)	0·158
Sensitivity Analysis 1^c^	1 [reference]	1·40 (0·92, 2·12)	2·23 (1·36, 3·66)**	<0·001***
Sensitivity Analysis 2^d^	1 [reference]	1·46 (0·96, 2·22)	2·14 (1·30, 3·54)**	0·001**
Sensitivity Analysis 3^e^	1 [reference]	1·90 (1·17, 3·08)**	2·32 (1·25, 4·32)**	<0·001***

Abbreviations: OR, odds ratios; CI, confidence interval.

a Adjusted for age, sex, baseline cognitive function, race, education, cohabitation status, sleep onset insomnia, sleep maintenance insomnia, daytime sleepiness, habitual sleep duration, depression, anxiety, diabetes, hypertension, stroke, self-rated health, physical inactivity, BMI, smoking status, alcohol intake, and medication use.

b Analysis stratified by sex, with adjustment for covariates in the fully adjusted model.

c Sensitivity analysis 1 further adjusting for doctor-diagnosed sleep disorders, in addition to covariates in the fully adjusted model.

d Sensitivity analysis 2 excluded participants who developed PD by the end of the follow-up period (*n =* 24). This analysis was adjusted for covariates in the full model.

e Sensitivity analysis 3 excluded participants with possible MCI at baseline (*n =* 515). This analysis was adjusted for covariates in the full model. *P < 0·05. ^⁎⁎^*P* < 0·01. ^⁎⁎⁎^*P* < 0·001.

In the secondary analyses stratified by sex, the associations between distressing dream frequency, cognitive decline, and incident dementia, were statistically significant amongst men but were non-significant amongst women (Tables 3 and 4).

In the sensitivity analyses, the associations were similar and remained significant after further adjusting for psychosocial stress in the middle-aged cohort (Table 3) and doctor-diagnosed sleep disorders in the older cohort (Table 4). The associations remained significant in both cohorts after excluding participants who developed PD by the end of the follow-up period (Tables 3 and 4). In the older cohort, the association was strengthened after excluding participants with possible MCI at baseline (Table 4).

## Discussion

In this large, prospective study of community-dwelling men and women without cognitive impairment or PD from the USA, a higher frequency of distressing dreams was positively associated with a higher risk of cognitive decline in middle-aged adults, and higher risk of incident all-cause dementia in older adults. These associations were independent of a wide range of possible confounders.

These findings are in line with several recent studies which showed that distressing dreams predict faster cognitive decline and dementia in people with PD.^4^, ^5^, ^6^ However, the present study extends these findings by demonstrating that this association is not specific to people with PD, and can be extrapolated to the general population.

These results are also consistent with a recent population-based longitudinal study in Finland, which identified that a higher frequency of nightmares during midlife, was negatively associated with MMSE scores during later-life (21–31 years later).^30^ However, given that this study did not assess cognition at baseline, it remained unclear whether midlife nightmares were a manifestation of pre-existing cognitive impairment, or whether they were associated with accelerated cognitive decline (a precursor of future dementia).^31^ As such, the present study extends these findings, by demonstrating for the first time that distressing dreams may predict accelerated cognitive decline in middle-aged adults - independent of baseline cognition.

This study is also the first to investigate the association between distressing dreams in community-dwelling older adults and the subsequent development of clinical dementia. Although, previous studies had evaluated cross-sectional associations.^32^ One of these studies showed that frequent nightmares were significantly more common amongst patients with dementia with Lewy bodies (DLB; 83.3%) and PD dementia (PDD; 77·7%)^32^ compared with general population estimates (2–6%),^1^^,^^2^^,^^7^ as well as other dementia syndromes (1–30%).^32^ Thus, one could speculate that distressing dreams in community-dwelling adults, might be most associated with the future development of a Lewy body dementia (DLB or PDD). This hypothesis would be consistent with the findings of an earlier longitudinal study, in a cohort of adults with confirmed MCI, which identified that having frequent nightmares was a stronger predictor of conversion to DLB (hazard ratio, 4.14), compared with Alzheimer's disease, vascular dementia or frontotemporal dementia.^33^ Furthermore, this hypothesis would be consistent with the recent finding that community-dwelling older adults with frequent distressing dreams, are at significantly increased risk of developing PD.^9^ However, given that Lewy body dementias accounts for less than 8% of dementias diagnosed in the community (DLB: 4·2%; PDD: 3·6%),^34^ and the fact that the associations were unchanged in this study after excluding possible PDD cases at follow-up (Tables 3 and 4), it therefore seems highly likely that distressing dreams may also predict the development of more common dementia syndromes, such as Alzheimer's disease - albeit to a lesser degree.

In the present study, the prevalence of frequent distressing dreams (“once a week or more”) was higher in the older cohort (6·9%), compared with the middle-aged cohort (6·0%). These findings are in line with earlier population-based studies, which identified that distressing dreams remain relatively stable throughout early adulthood,^2^ but then progressively increase in prevalence from middle to older adulthood.^2^^,^^7^ The results of the current study suggest that the emergence of distressing dreams during middle and older adulthood, may represent early signs of a neurodegenerative dementia in some individuals.

Intriguingly, population-based cross-sectional studies have repeatedly shown that women are more likely than men to experience frequent nightmares between adolescence and middle adulthood,^35^ yet from around age 65 onwards,^2^^,^^7^^,^^35^ there is no longer a gender gap in nightmare frequency - as men eventually ‘catch up’ with women.^2^^,^^7^ Interestingly, a similar pattern was seen in the present study. The prevalence of frequent distressing dreams was 4·1% amongst the middle-aged men, which rose to 8·5% in the older men (107% increase). Whereas the prevalence of frequent distressing dreams was 7·4% amongst the middle-aged women, but unexpectedly decreased to 5·7% in the older women (23% decrease). Moreover, even though the prevalence of frequent distressing dreams increased amongst older men and older women, between baseline and follow-up (older men: +1·9%; older women: +1·2%); the absolute change in prevalence was nevertheless higher amongst men. Thus, if distressing dreams emerging during middle to older adulthood represent early signs of a neurodegenerative dementia in some individuals, we should expect a stronger association between distressing dream frequency and later cognitive outcomes amongst community-dwelling men, as compared with community-dwelling women (given that men are more likely than women to develop distressing dreams during middle and older adulthood).

As expected on this hypothesis, the results from the sex-stratified analyses showed that distressing dreams were strongly and statistically significantly associated with cognitive decline and dementia amongst the men (Tables 3 and 4), but were only weakly and non-significantly associated with cognitive decline and dementia amongst the women (Tables 3 and 4). As such, these findings are compatible with a neurodegenerative theory of adult-onset distressing dreams.^9^ Moreover, given that depression was also found to be associated with incident dementia in this study (aOR = 1·89; 95% CI: 1·17-3·04) – as has been observed in many previous studies^22^ – it may well be the case that distressing dreams and depressive symptoms in the preclinical phase of dementia, are both caused by neurodegeneration of right frontal brain regions, that are required for downregulating negative emotions across conscious states (i.e., dreaming and wakefulness).^36^ This theory would be in line with the neurocognitive model of nightmares.^37^

An alternative hypothesis, which merits some discussion based on these findings, is the possibility that the association between distressing dreams and future cognitive impairment, is driven by adults with co-existing idiopathic REM sleep behaviour disorder (iRBD). This hypothesis has some plausibility, given that polysomnography-confirmed iRBD is a known prodrome of DLB and PDD,^38^ is more common in men than women,^38^ and is strongly associated with distressing dreams (one third of iRBD patients experience nightmares weekly).^39^ On the other hand, a significant proportion of patients with RBD experience vivid, but non-frightening dreams – and some do not remember their dreams at all. Furthermore, the fact that further adjusting for doctor-diagnosed sleep disorders had no effect on the associations in this study (Table 4), seems to argue against this hypothesis. Although, this does not rule out the possibility that the associations might have been driven by participants with undiagnosed iRBD. However, this too seems unlikely, given that iRBD is believed to be a rare condition (<1% prevalence in these age groups)^40^ and the fact that previous population-based cohort studies found no association between probable iRBD and the subsequent development of all-cause dementia.^41^ Finally, given that distressing dreams predict cognitive decline and dementia in people with PD - irrespective of whether or not they have RBD^4^, ^5^, ^6^; it therefore seems likely that this would also be the case in community-dwelling adults without PD.

It is also noteworthy that there was a linear relationship between distressing dream frequency and rates of cognitive decline and dementia; in both the middle-aged cohort (Table 3) and the older adult cohort (Table 4). This suggests that that a higher frequency of distressing dreams may represent a more advanced stage of neurodegeneration. Indeed, this theory would be in line with a recent cross-sectional study, in 28 patients with nondemented PD, which identified that their self-reported distressing dream frequency, was positively correlated with atrophy of grey and white matter in their frontal lobes.^42^ Moreover, a recent longitudinal study in 23 patients with nondemented PD, showed that the more frequently patients reported having dreams containing negative emotions at baseline, the faster their global cognitive function declined during a 4-year follow-up period.^5^

As such, the results of the present study, alongside the aforementioned studies, suggest that screening for distressing dreams in the general population may help to identify individuals in the earliest stages of a neurodegenerative dementia - several years or decades before a clinical diagnosis. Moreover, by tracking changes in distressing dream frequency over time, this may even allow doctors to determine how close these individuals are to the onset of clinical dementia. Given that the first-line pharmacological treatment for nightmares (prazosin) has been shown to prevent memory decline and reduce amyloid β generation in preclinical studies of Alzheimer's disease^43^ – this raises the possibility that treating distressing dreams might even help to slow cognitive decline and prevent cognitive impairment.

This study has several strengths, including the prospective design, long follow-up period, use of a well validated questionnaire for assessing habitual sleep disturbances, inclusion of a wide range of potential confounders, and the community-based approach involving three independent cohorts. Furthermore, the participants were not selected based on distressing dream frequency or cognitive status. The study does have limitations however. First, as with most large population-based cohort studies, there was no clinical dementia adjudication or information on subtypes of dementia. Second, the questionnaire item used to assess distressing dreams does not clearly distinguish between bad dreams (i.e., distressing dreams without awakenings) and nightmares (i.e., distressing dreams with awakenings). As such, it is not possible to determine whether the associations with later cognitive outcomes may vary by distressing dream subtype. Also, many people in the general population have negatively toned dreams which they may not consider to be bad dreams or nightmares. Given that the term ‘bad dreams’ was not defined for participants in this study, it is possible that they may have responded to the questionnaire item in different ways. Third, given that the questionnaire item focused on distressing dreams that cause difficulty sleeping, there remains a possibility that the associations were not due to the distressing dreams themselves – but rather the sleep disturbances caused by them. However, given that the associations were similar – or even strengthened - after adjusting for sleep onset insomnia (difficulty falling asleep) and sleep maintenance insomnia (difficulty staying asleep), this strongly suggests that the associations were due to the dreams themselves, and not the disturbances caused by them. Fourth, although the use of antidepressants, benzodiazepines and hypnotics were adjusted for in the analyses, it remains possible that another medication group - or use of these medications earlier on in life - may have biased the associations. Fifth, it is possible that including covariates with missing data might have introduced some bias. However, the results were similar and remained significant after including only participants with complete covariate data (not shown). Sixth, it is possible that some patients with MCI were still included in the study sample despite the baseline exclusions. However, the results were even stronger in the older cohort after excluding those with possible MCI based on baseline cognitive scores (Table 4). Interestingly, the association between distressing dreams and incident dementia even became statistically significant amongst older women after excluding these participants (data not shown). As such, this suggests that the inclusion of individuals with MCI – possibly leading to diminished dream recall ability^44^ - might have actually led to an underestimation of the associations, especially amongst women.^44^ Finally, given that the study included mostly white men and women, it is possible that the findings might not be generalisable to more racially diverse populations. However, this limitation was mitigated by controlling for race in the analyses.

In summary, this study provides evidence for the first time that a higher frequency of distressing dreams in community-dwelling adults without cognitive impairment or PD, is positively associated with faster cognitive decline during midlife, and increased risk of developing dementia during later-life. As such, this study suggests that screening for distressing dreams in the general population may help to identify individuals in the preclinical phase of dementia,^31^ in whom early interventions to prevent cognitive impairment could be targeted.^22^^,^^23^^,^^43^

## Contributors

AIO was responsible for conception, organisation, and execution of the research project; design and execution of the statistical analysis; verification of the underlying data; and manuscript preparation. AIO had full access to all the data in the study and accepts responsibility for the decision to submit for publication.

## Data sharing statement

Data from MIDUS are available at: https://www.icpsr.umich.edu/web/NACDA/series/203. Data from SOF are available at: https://sofonline.ucsf.edu. Data from MrOS are available at https://mrosonline.ucsf.edu. The analysis dataset for this specific manuscript is also available from the corresponding author upon request.

## Declaration of interests

The author declares no conflict of interest.

## Appendix Supplementary materials

Image, application 1
